# Supplementary material for: Are carbon emissions trading and green financial instruments synergistic? -Comprehensive quantitative research based on content analysis
Source: PLoS One. 2024 Mar 7;19(3):e0298601. doi: 10.1371/journal.pone.0298601 (PMC10919601; doi:10.1371/journal.pone.0298601)
Supplement: S1 Data — (ZIP) [file pone.0298601.s001.zip › Data/地级市Z_F工作报告.zip!/║╙─╧/Σ≡║╙╩╨/└┤╫╘╣1⁄2╓┌║┼í╛┬φ┐╦╩2╛▌═°í┐_2014.docx]

各位代表：

现在，我代表市人民政府，向大会报告工作，请予审议，并请各位政协委员和其他列席人士提出意见。

一、2013年工作回顾

过去的一年，市政府在省委、省政府和市委的正确领导下，在市人大、市政协的监督支持下，深入贯彻落实科学发展观，团结带领全市人民，凝神聚力，攻坚克难，奋勇争先，经济社会发展取得新的成效。全市生产总值861.5亿元，增长9.4%;固定资产投资648.3亿元，增长23.1%;社会消费品零售总额336.1亿元，增长14.1%;城镇居民人均可支配收入21174元、农民人均纯收入9876元，分别增长10.7%和12.8%。

——经济运行质量明显提高。全市规模以上工业实现利润270亿元，居全省第6位;财政总收入突破百亿元大关，比上年增加16.9亿元;地方公共财政预算收入53.9亿元、比上年增加12.4亿元，增长29.7%、增速居全省第2位;税收收入43.7亿元，占地方公共财政预算收入的比重达到81%、居全省第1位。

——产业结构调整步伐加快。食品产业支撑作用更加明显，实现主营业务收入1435亿元、利润177.9亿元，分别占全市规模以上工业的64.2%和65.9%。电子信息、生物医药等战略性新兴产业发展态势良好。城区工业企业“退二进三”、“退城入园”力度加大，关停拆除了8家高耗能、低效益企业。服务业发展水平得到提升，实现地税收入23.4亿元，比上年增加6.3亿元，占全市地税收入的比重达到64.6%。

——发展动力和后劲持续增强。“全国质量强市示范城市”创建工作扎实推进，全省首家“国家肉及肉制品检测重点实验室”通过验收，双汇集团荣获省长质量奖。新认定国家级高新技术企业8家、省级工程技术研究中心4家。38家企业实施了战略重组，22家企业在深圳前海股权交易中心挂牌交易。开放招商成果丰硕，双汇国际成功并购美国史密斯菲尔德公司;成功举办了第十一届中国(漯河)食品博览会，中国食品名城的知名度和影响力进一步扩大;全市实际利用外资7亿美元、居全省第4位，实际利用省外资金170亿元、增长26.7%。争取上级财政性资金88.2亿元，比上年增加14.8亿元。150个市定重点项目完成投资225.3亿元，59个项目建成投运。产业集聚效应更加明显，全市6个产业集聚区实现主营业务收入1500亿元，从业人员达到15.4万人。

——“三农”工作再创佳绩。投入和发放各类涉农资金26.3亿元。累计建成高标准粮田72.7万亩，粮食总产173.6万吨、实现“十连增”。蔬菜种植面积达92.5万亩。完成植树造林面积4.5万亩。新培育省级农业产业化集群8个、年销售收入超千万元的农业产业化龙头企业19家，新增农民专业合作社726家，组建了全省首家大型畜牧合作社，我市成为全省生态畜牧业建设试点市。农村土地流转面积突破100万亩，占家庭联产承包经营总面积的43.7%、居全省第3位。全市耕种收综合机械化水平达到83%，秸秆禁烧和综合利用工作取得历史最好成绩。南水北调配套工程快速推进。农田水利和农村基础设施建设力度加大，新发展有效灌溉面积12万亩、旱涝保收田10万亩，新解决22万人的饮水安全问题，新建户用沼气池3380座，新建、改造农村公路145公里。新改造农村危房5446户。完成扶贫开发整村推进项目20个，实现2.5万农村贫困人口稳定脱贫。新型农村社区建设稳步推进，累计建成住房2.8万套，7800多户入住新居。

——城市建设扎实推进。新修编的城市总体规划获省政府批准，41项城市专项规划编制加快推进。沙澧河开发建设一期工程持续完善提升、二期工程方案基本确定。新建、改造城市道路32条，107国道漯河段改造工程、241省道漯河段改建工程、交通路沙河桥拓宽改造工程竣工通车，人民路火车站区域地下通道建成投用，解放路沙河桥、太白山路沙河桥开工建设。旧城区和城中村改造实现了历史性突破，征收房屋面积285万平方米，启动安置房建设面积248万平方米。供气、供水、排水等基础设施和环卫设施建设力度加大，市区旱厕全面消除，市污泥处置厂建成投用。城乡一体化示范区(漯河新区)总体发展规划、起步区控制性详细规划通过专家评审，西城区管理体制得到理顺、开发建设加快推进，商务中心区和特色商业区发展规划编制全部完成。城市公交改革攻坚破难，组建了国有控股公交集团，配置、更新了115台新型节能环保公交车辆，新开通了3条公交线路。完成了市区5个农贸市场升级改造、10个老旧小区综合整治，物业管理、违法建设等热点难点问题得到有效解决。创建国家卫生城市工作深入推进，专项整治成效显著，城市环境明显改善，城市整体形象得到进一步提升。

——民生质量显著提高。全市各级财政用于民生的支出达到90.9亿元，占地方公共财政预算支出的72.5%;省定十项民生工程、市定15件民生实事较好完成。全市城镇新增就业5.6万人、再就业2万人，新增农村劳动力转移就业4.1万人。基本医疗保障制度实现全覆盖，企业退休人员基本养老金、城镇居民医疗保险大病补偿、新农合大病补偿、城乡居民最低生活保障标准、农村五保供养标准全面提高。社会养老服务体系、社会救助体系和社会福利体系进一步完善。发放个人住房公积金贷款6.9亿元，新开工建设各类保障性住房1.5万套，我市被确定为全省唯一的住房保障创新试点城市。

社会事业全面进步。全市新建、改扩建幼儿园106所，市区新建、续建、改扩建中小学9所;普通高考本科上线人数继续保持在万人以上;职业教育集团化发展步伐加快。公立医院改革稳步推进，国家基本药物制度初步建立，县区医院病房楼和乡镇卫生院、村卫生室改扩建等一批项目建成投用，城乡医疗卫生服务体系进一步完善。我市连续六次荣获“全国无偿献血先进城市”称号。文化旅游业加快发展，成功举办了纪念贾湖遗址发掘30周年暨贾湖文化国际研讨会，舞阳农民画入选全国“讲文明、树新风”公益广告，“周末沙澧文化超市”、“幸福漯河健康舞”荣获全省首届群星奖，许慎文化园续建工程竣工，周彦生艺术研究院二期开工建设。举办了16项省级以上体育赛事，全民健身运动广泛开展。计划生育、环境保护和土地管理三大基本国策全面落实，生态文明建设成效明显，我市被授予全省环境保护目标完成优秀单位。统计、审计、人防、气象等工作进一步加强，广播电视、新闻出版、外事侨务、民族宗教、史志档案、慈善事业等取得新成效，妇女、未成年人、老年人和残疾人合法权益得到保障。国防教育、双拥工作继续加强，军民融合式发展进一步深化，驻漯解放军、武警消防官兵和民兵预备役人员在支持地方建设中作出了重要贡献。

社会大局和谐稳定。民主法制建设不断加强，自觉接受人大和政协监督，人大代表建议和政协提案办理质量进一步提高，依法治市和法治政府建设扎实推进。廉政建设力度加大，政风行风进一步好转。精神文明建设成效显著。信访和安全生产形势持续平稳，食品药品监管得到强化，平安漯河建设深入推进，应急处置能力明显提高，公众安全感指数居全省前列。我市荣获全国法治城市创建工作先进城市、全国社会管理综合治理工作优秀市、全省信访工作先进市、全省安全生产优秀市等称号。

各位代表!过去的一年，面对严峻形势和繁重任务，我们积极应对、科学谋划，勇于负责、敢于担当，在转型中创新思路、在挑战中坚定信心、在克难中加快发展。始终做到咬定目标不放松，深入研判形势，加强运行监测，全力保增长，持续调结构，加快促转型，全面提高质量效益;始终做到狠抓重点强推进，深化企业服务，现场解决问题，开展百日攻坚，强化督导问效，有力推动工作落实;始终做到创新举措求突破，面对各种难题，勇于攻坚克难，健全规划管理体制，完善城市“两改”政策，创新项目推进机制，加大银企对接力度，拓宽多元融资渠道，在改革创新中破解发展难题;始终做到尽心竭力惠民生，做决策、办事情，充分听取群众意见、充分考虑群众利益，在任何情况下都把改善民生放在心上、抓在手上、落实在行动上，尽最大努力提升民生质量;始终做到上下同心聚合力，各县区竞相发展，各部门顾全大局，各级领导率先垂范，广大干群合力攻坚，全市上下在团结拼搏、务实重干中创造了新业绩、铸就了新辉煌。

各位代表!过去的一年，在宏观形势错综复杂、区域竞争日益激烈、结构调整任务艰巨、经济下行压力加大的情况下，我们能取得这些成绩确实不易。这是省委、省政府和市委正确领导的结果，是市人大、市政协监督支持的结果，是全市人民团结拼搏的结果。在此，我代表市人民政府，向工作在各条战线上的工人、农民、知识分子、干部，向驻漯解放军、武警消防官兵、公安民警及各界人士，致以崇高的敬意!向所有关心支持漯河发展的同志们、朋友们，表示诚挚的感谢!

回顾过去一年的工作，我们也清醒地认识到，全市经济社会发展还面临不少困难和挑战，政府工作与自身建设还存在不少差距和不足。主要表现在：全市经济产业层次总体不高，高新技术产业规模小，科技创新能力不强，骨干企业群体不大，服务业仍是全市经济发展中的短板;县区经济发展需要进一步加快;城镇化水平不高，城镇功能不够完善;资源环境约束加剧，融资、用工等瓶颈制约依然存在;民生改善和社会事业发展与群众期望还有一定差距;政府职能转变还不到位，作风不实、服务不优、效能不高等问题仍不同程度存在，一些公务人员能力素质还不适应新形势、新任务要求，极个别部门人员群众观念淡薄、服务意识较差，不作为、慢作为、乱作为现象时有发生，严重损害了政府形象。对此，我们一定高度重视，认真对待，采取有力措施，切实加以解决。

二、2014年工作安排

今年是全面完成“十二五”规划的关键一年。政府工作的总体要求是：全面贯彻落实党的十八届三中全会和省委、市委经济工作会议精神，以稳中求进、进中提升为总基调，以六项重大攻坚任务为统揽，以项目建设为中心，以作风改进为保证，着力稳增长、调结构、抓转型、促改革、惠民生，努力保持经济持续健康发展、社会大局和谐稳定。

今年经济社会发展的主要预期目标是：生产总值增长10%左右，地方公共财政预算收入增长12%，固定资产投资增长22%左右，社会消费品零售总额增长14%，城镇居民人均可支配收入、农民人均纯收入增长10%，城镇新增就业4万人，人口自然增长率控制在省定目标以内，省定节能减排目标全面完成。

实现上述目标，要重点抓好八项工作。

(一)围绕招大引强，着力提高开放招商水平。坚持以开放招商为统领，紧盯世界500强、国内500强、中央企业和行业龙头，深入推进产业链招商和集群招商，力争招引一批重大项目。创新招商方式。突出以商招商，充分发挥企业的招商主体作用，引导企业与境内外大企业、大财团进行战略合作;鼓励现有企业进一步增资扩能，拉长产业链条，加快产业集聚。突出招商重点。紧紧围绕各县区、城乡一体化示范区、经济技术开发区、西城区的产业定位，强力攻坚招商，努力在龙头企业和重大项目招引上实现新突破。尤其是对全市发展具有战略意义的食品、电子信息、生物医药等专业园区和商务中心区、特色商业区，招商引资要大见成效。精心办好第十二届中国(漯河)食品博览会，持续扩大中国食品名城的知名度和影响力。加大开放力度。拓宽开放领域，加快服务业、现代农业、基础设施和社会事业等领域开放，力争在银行保险、现代物流、电子商务、文化旅游、科技、医疗、教育等领域引进一批合作项目。毫不动摇鼓励、支持、引导非公有制经济发展，大力引进外来资本和民间资本。

(二)围绕重点领域，着力抓好项目建设。坚持以项目为抓手、用项目作支撑，以项目大建设带动大投资、推动大发展。今年，安排市级重点项目150个，完成投资300亿元以上，带动全市固定资产投资完成800亿元以上。狠抓重点领域项目。加快推进中大天然绿色科技产业园、兴茂钛业氯化法钛白粉、裕松源药业生物医药产业园、西盟·健客乳品工业园、中盐公司真空制盐等一批即将竣工投产的项目，开工建设旺旺新工业园、双汇第二工业园二期、嘉吉(临颍)工业园、渴望通讯等一批新项目，大力支持现有企业新上项目、扩大产能;着力抓好服务业、基础设施、民生事业等一批重点项目，全面提升重点项目质量和效益。完善项目推进机制。坚持重点项目推进协调例会、各级领导分包代理服务、重点项目联审联批、定期观摩讲评等制度，实行项目建设专项述职制度，推动全市重点项目建设实现新突破、再上新台阶。

(三)围绕转型升级，着力推动工业提速增效。调整优化工业经济结构。坚持做强食品工业，以双汇国际食品产业园、中国食品百强工业园、中国(漯河)食品包装机械产业园、临颍休闲食品产业园为主要载体，强化食品工业战略支撑作用，加快建设全国最具竞争力的中国食品名城。坚持做大新兴产业，着力抓好东兴电子产业园、漯河健康产业园、沙澧医疗器械产业园建设，加快建设全省重要的电子产品生产交易基地、生物医药生产基地。坚持做优传统产业，整合资源，统一布局，扩大高端化工产品规模，加快建设舞阳高端盐化工产业基地;加快推动机械、造纸、制鞋、纺织等传统产业转型升级，使之焕发新的生机与活力。加快培育骨干企业群体。在全市选定30家重点企业、50家中小企业、100家小微企业，全力重点培育，经过几年努力，形成大型企业引领、中型企业支撑、中小企业快速提升跟进的发展格局。今年，全市新增年销售收入超亿元企业30家以上、超10亿元企业4家、超40亿元企业2家。持续提升产业集聚区发展水平。按照产业集聚区功能定位，分类入驻项目，拉长产业链条，加快产业集聚。节约集约利用土地，切实提高投资强度，健全完善服务功能，推动产城融合发展，进一步增强产业集聚区发展竞争力，争创全省示范产业集聚区，努力在全省产业集聚区考核中位次前移。进一步强化企业服务和技术创新。完善服务企业长效机制，及时解决企业生产经营中遇到的困难和问题。深化“科技创新三年培育计划”，实施创新型企业培育工程，加快企业研发中心建设，支持企业组建产业技术创新联盟，积极推进工业化和信息化融合，推动企业技术进步和产业结构优化升级。

(四)围绕现代农业发展，着力做好“三农”工作。以创建全国现代农业示范区为目标，全面落实强农富农惠农政策，切实加大“三农”投入，加快提升农业和农村发展水平。持续抓好高标准粮田建设。加大涉农项目整合力度，统筹推进水、电、路、林等田间生产设施建设，新建高标准粮田36.3万亩。重点抓好舞阳泥河洼16万亩高标准粮田建设，努力打造成全省样板、全国典型;加快推进临颍、郾城两个10万亩高标准粮田建设。全年粮食总产稳定在170万吨以上，把漯河打造成为全省粮食优质高产示范市。加快培育现代农业产业化集群。重点抓好畜牧养殖及加工、果蔬加工、面制品、乳品等现代农业产业化集群，全市新建市级现代农业示范园区10个以上，新培育优势农业产业化集群6个以上。着力培育农业产业化龙头企业，发挥其示范带动作用。加快发展都市生态农业，在城区周边乡镇，以特色种植业、设施园艺业、生态休闲业等为主体，规划建设兼具城市“菜篮子”、生态绿化、休闲观光等功能的现代农业园区。深入推进双汇产业化工程提升行动，实施生态畜牧业建设工程，积极创建全省生态畜牧业示范市。着力构建新型农业经营体系。建立完善农村土地流转服务体系，规范土地流转程序，扩大土地流转规模。大力培育专业大户、家庭农场、农民合作社、农业企业等新型农业经营主体，农民专业合作社发展到2000家、家庭农场达到150家。培育有文化、懂技术、会经营的职业农民。完善现代农业技术体系、农业科技推广体系和农业社会化服务体系，加强优良品种繁育和推广，提高农业物质技术装备水平，争创全国农业机械化示范市。加快气象现代化建设，搞好气象服务。积极发展农业保险，提高农业生产抗风险能力。完善补贴制度，提高补贴精准性、指向性。强化生产源头治理和产销全程监管，确保农产品质量安全。扎实推进农村基础设施建设。抓好农田水利建设、中小河流域治理和南水北调配套工程建设，新打机井3000眼、维修机井6000眼，新解决20万人以上的饮水安全问题，新建农村户用沼气池2500座，新建、改造农村公路150公里以上，完成扶贫开发整村推进项目16个以上，进一步改善农村生产生活条件。

(五)围绕城乡统筹发展，着力加快新型城镇化建设。不断优化城镇布局，积极构建以中心城市为核心、两个县城为组团、重点镇为节点的现代城镇体系。完善城乡规划体系。全面完善提升城市各类专项规划，建设规划信息“一张图”管理系统，以县区为单元，完善修订村镇规划，实现城乡建设规划全覆盖。高标准编制规划，严格落实规划，确保规划的科学性、权威性和严肃性，充分发挥好规划的龙头作用。加大中心城市建设力度。围绕打造独具魅力的美丽漯河、生态宜居的幸福漯河、充满生机的活力漯河、集聚人气的实力漯河，进一步完善城市功能、丰富城市内涵、提升城市品位。积极稳妥推进旧城区和城中村改造，征拆房屋面积470万平方米，完成安置房建设360万平方米。开工建设西城区金融商务中心，启动开发改造中华世纪城、老寨里等街区，着力打造沙澧河交汇处标志性建筑、双汇总部基地、荣昌商业广场等重要节点，凸显城市个性和特色。重点抓好中山路上跨立交桥、沙澧连通桥闸工程、城市地下排水等十大城建工程，切实抓好保障房建设、百座街头健身游园建设、百个老旧小区整治等十项惠民工程，不断改善人居环境。深入推进城市公交场站建设等其它城建工程。加快城乡一体化示范区开发建设，强力实施起步区基础设施三年行动计划。优化经济技术开发区发展规划，加快基础设施和配套服务设施建设，着力打造软硬环境，提升国家级开发区形象。紧盯“三年出形象、五年成规模、八年见新区”发展目标，加大西城区开发建设力度，全年完成投资30亿元以上。坚持高标准、严要求、快推进，持续加大创建国家卫生城市工作力度，实现城市形象大提升。推动县城提速提质发展。加快临颍、舞阳两个县城老城区改造、新城区建设和产业集聚区建设，培育各具特色的支柱产业，主动承接中心城市产业辐射，吸纳农村人口转移，形成老城区、新城区和产业集聚区互动发展格局。积极发展重点镇。抓好中心城区周边卫星镇建设，将其作为中心城区扩展区域，在镇域规划、基础设施、社会保障等方面加强对接，逐步实现融合式发展。发挥中心镇、特色镇的产业、区位等优势，培育特色产业，完善城镇功能，加快人口集聚。扎实推进新农村建设。按照产业、新农村、土地、公共服务和生态规划“五规合一”要求，编制修订完善新农村规划。城中村、城郊村和产业集聚区内的村庄，统一纳入城市规划、建设和管理。以乡镇政府所在地的村庄和特色经济强村为重点，因地制宜，分类推进新农村建设。对新型农村社区建设，根据情况，区别对待，已经建成的、引导群众入住，正在建设的、持续健康推进，尚未开工的、深入科学论证，充分尊重群众意愿，达到群众满意。加强生态文明建设。进一步加大大气污染和水污染防治力度，有效遏制雾霾污染，不断改善水环境质量。加大力度抓好“乡村清洁工程”，加强农村面源污染治理和农村环境综合整治，开展美丽乡村创建，改善农村人居环境质量。扎实推进林业生态市建设，大力提升城乡绿化一体化水平，加快建设美丽漯河。

(六)围绕规模膨胀，着力发展服务业。瞄准打造区域性现代商贸物流中心目标，加快构建与主导产业相配套、与城镇化进程相协调、与城乡居民需求相适应的服务业体系，推动全市服务业发展提速、比重提高、水平提升。深入推进商务中心区和特色商业区规划建设。把“两区”作为推动服务业快速发展、集聚发展的重要载体，加大招商引资力度，加快基础设施建设，完善配套服务功能，力争早日把“两区”培育成全市服务业发展的增长极。大力发展现代物流和商贸业。着力抓好一批物流、商贸业重点项目，沙北丹尼斯百货广场、豫中南农产品批发市场等项目建成投运，加快推进人民路城市综合体、荣昌商业广场等一批城市综合体项目，开工建设沙河港物流园区、中农物流公司公铁联运、国家公路运输枢纽甩挂物流园、豫中南(国际)农机机械装备产业城、果品批发市场等项目，抓好进口肉类指定口岸漯河查验区建设。发挥区位优势，整合要素资源，着力培育冷链、粮食、汽贸、家电及电子消费品等物流品牌。推动重点市场扩规模、快提升、上水平，进一步增强辐射带动能力。繁荣发展文化旅游业。以许慎文化、贾湖文化、舞阳农民画、沙澧文化超市等为重点，打响特色文化品牌。健全完善公共文化服务体系，加快推进市图书馆、博物馆、群艺馆、城市展示馆等项目建设，建成投用河上街古镇等项目，提升完善沙澧河风景区、许慎文化园、开源金凤凰文化产业园、周彦生艺术研究院、小商桥等景区的品位和功能。积极发展金融等服务业。抓好金融服务业，积极引进股份制商业银行在我市设立分支机构，支持各商业银行发展，加快推进农村信用社改革，支持村镇银行做大做强，促进担保公司、小额贷款公司健康发展。切实加强银企对接工作，着力破解企业融资难题。抓好信息服务业，持续深化电子政务、电子商务及信息网络技术在经济社会领域的全方位应用。抓好房地产、社会养老、商务会展等服务业，全面提升服务业发展水平。

(七)围绕改革创新，着力激发动力活力。按照上级部署，结合我市实际，努力推动重点领域、关键环节的改革取得新进展。深化行政体制改革。进一步推进简政放权，继续清理行政审批事项和具有审批性质的管理事项，该放的放到位，该批的简化程序，该管的管住管好。优化政府机构设置、职能配备，全面完成政府机构改革任务。严格控制机构编制。加快事业单位分类改革。深化财税体制改革。深入推进政府和部门预决算公开。清理整合专项转移支付项目，统筹整合专项资金，提高财政资金使用效益。清理规范税收优惠政策，全面落实营改增等改革政策。加强政府债务管理，提升投融资公司的投融资能力、资产管理能力和风险防范能力。深化土地管理和户籍制度改革。加快农村集体建设用地和宅基地确权登记颁证工作，消除农民进城后顾之忧。全面放开市域内落户限制，促进有能力在城镇稳定就业和生活的常住人口、已经转移到城镇就业的农业转移人口有序实现市民化。深化社会事业领域改革。深化医药卫生体制改革，抓好县级公立医院综合改革，巩固完善基本药物制度。深化科技、教育、文化等社会事业领域的改革，全面释放改革红利，为全市经济社会发展注入新的动力和活力。

(八)围绕民生改善，着力提升群众幸福指数。坚持把保障和改善民生作为政府工作的出发点和落脚点，投入更多的财力、付出更大的精力、采取更有力的举措，扎实做好民生工作，大力发展民生事业，全面完成省定民生工程，认真办好市定民生实事，尽最大努力让发展成果更多更公平地惠及全市人民。加大就业工作力度。实施更加积极的就业政策，健全城乡就业服务体系，深入推进全民技能振兴工程，重点做好高校毕业生、农村转移劳动力、城镇困难人员、退役军人就业工作。完善扶持创业优惠政策，充分发挥创业带动就业的倍增效应。完善社会保障体系。提高企业退休人员基本养老金、城乡低保补助标准、农村五保供养标准和优抚对象补助水平，积极推动城镇企业职工基本养老保险与城乡居民社会养老保险衔接，提高新农合筹资标准，对农村居民实行大病保险，加快实施社会保障“一卡通”。高度重视养老产业发展，切实抓好全国养老服务业综合改革试点工作，开工建设市养老产业综合服务园，加快城市社区日间照料中心、农村幸福院等养老设施建设，积极探索医养结合养老模式，新增养老床位1000张以上。加大保障性住房建设力度，新开工各类保障性住房2.3万套。完善住房公积金制度，帮助更多职工改善居住条件。

加快发展社会事业。深入推进科教兴漯和人才强市战略，加大人才引进、培养力度。全方位提升质量水平，确保成功创建全国质量强市示范城市。坚持优先发展教育事业，建成投用2所市区中学，改扩建市直2所中学，在市区和临颍县城筹建7所中小学，开工建设青少年综合实践基地;全市143所中小学达到义务教育均衡发展标准;在全市初中和小学启动“数字校园”工程建设，推进信息技术与教育教学深度融合，促进优质教育资源普及共享;继续保持高中教育在全省的领先位次;大力发展高等教育和职业教育，支持漯河医专升本和职业技术学院提升转型发展，加快技师学院新校区、食品职业学院扩建和第一中专搬迁等项目建设。完善基本医疗卫生服务体系，抓好市中心医院西城区分院、全科医生临床培养基地、市第二人民医院扩建、市第六人民医院病房楼、市康复医院、县区医院和急救中心、薄弱乡镇卫生院改扩建等项目建设。继续抓好重大传染病防控救治工作。从今年起，利用三年时间，对全市2000名基层卫生人员进行全面培训。举办高水平体育赛事，争创全省全民健身示范城市。关心妇女、儿童、残疾人、老龄、慈善等事业发展，建设100个农村留守儿童之家，建成投用市残疾人康复中心。认真做好第三次全国经济普查工作。继续做好统计、广播电视、新闻出版、外事侨务、民族宗教、史志档案等工作。调整完善生育政策，促进人口长期均衡发展。支持工会、共青团、妇联等人民团体发挥桥梁纽带作用。加强国防教育和国防后备力量建设，支持驻漯解放军、武警部队、预备役部队、民兵和人民防空建设，推动军民融合深度发展。深入开展双拥共建，巩固军政军民团结。

加强和创新社会管理。高度重视社区建设，健全基层综合服务管理平台和矛盾纠纷调处机制。完善和创新流动人口、特殊人群管理服务机制。做好第八届村委会换届选举工作。大力培育社会主义核心价值观，深入推进公民道德建设，广泛开展群众性精神文明活动，提升全民文明素养。认真做好信访稳定工作，完善社会稳定风险评估机制，切实抓好安全生产和食品药品安全监管，不断深化平安漯河建设，强化社会管理综合治理，加强应急管理体系建设，严厉打击各类违法犯罪活动，确保全市社会大局和谐稳定。

三、切实加强政府自身建设

面对复杂的宏观形势、繁重的发展任务、群众的殷切期盼，必须更加注重政府自身建设，按照市委统一部署，扎实开展党的群众路线教育实践活动，全面转作风、提效能、优服务、争一流，提振精气神，汇聚正能量，务求大作为，努力建设人民满意的政府。

坚持服务为先，认真履行职责。始终把群众利益放在首要位置，把人民满意作为第一标准，加快转变政府职能，不断优化服务流程，全面提高服务水平。集中整治企业周边环境和重点项目建设环境，深入开展“双整双促双承诺”、涉企涉民科室评议、“百姓问政”等活动，认真做好市长热线和政风行风热线办理工作，着力打造更加优良的发展环境。牢记为民宗旨，全力服务群众，感同身受体验群众困难，设身处地考虑群众需求，推己及人谋求群众福祉，通过一件件小事的办理、一件件实事的落实、一件件难事的解决，赢得人民群众的更多信任，获得人民群众的更大支持。

坚持实干为要，弘扬担当精神。一分部署，九分落实。大力弘扬“钉钉子”精神，以踏石留印、抓铁有痕的作风啃下“硬骨头”，以不达目的誓不罢休的劲头打好“攻坚战”。强化效能意识，细化“任务分解表”，制定“推进路线图”，以目标倒逼进度，以督查强化落实，推动政府工作提速提效。深入一线，靠前指挥，雷厉风行干事业，身先士卒作表率。敢于担当、勇于创新，面对困难不退缩、遇到矛盾不回避，始终保持干事创业的热情、动真碰硬的豪情、奋发有为的激情，不断创造经得起实践、群众和历史检验的业绩。

坚持责任为重，切实转变作风。认真落实中央八项规定和省委、市委贯彻意见，从人民群众最需要的地方做起，从人民群众最不满意的地方改起，认真解决“四风”突出问题，推进作风建设常态化、长效化。大兴调查研究之风，眼睛向下看，身子往下沉，走进一线察民情，深入基层接地气。坚持求真务实，有面子、没里子的事不干，只顾眼前、不顾长远的事不干，与民争利、引起民怨的事不干。精简会议文件，改进会风文风，减少往来应酬，把更多的精力用在抓工作落实上、用在为群众解决实际问题上、用在加快漯河发展上。

坚持法治为上，严格依法行政。坚决执行人大及其常委会决议决定，自觉接受人大法律监督、工作监督和政协民主监督，认真办理人大代表建议和政协提案，广泛听取民主党派、工商联、无党派人士和人民团体意见。切实加强行政监察和审计监督，虚心接受社会监督和舆论监督。坚持依法科学民主决策，完善重大决策合法性审查和风险评估机制。提高行政执法水平，完善行政复议制度，深化法制宣传教育，做好法律援助工作。深入推进政务公开，向群众说真话、交实底，让百姓看得懂、说得清，创造条件让人民监督政府、让权力在阳光下运行。

坚持清廉为本，做到廉洁从政。始终敬畏手中的权力、敬重服务的人民，筑牢思想防线、守住廉洁底线，干干净净干事、清清白白为官。严格落实“一岗双责”，切实加强廉政教育，反腐倡廉常抓不懈，拒腐防变警钟长鸣，坚决纠正损害群众利益的不正之风，严厉查处各类违纪违法行为。坚持从严从俭，在任何时候、任何条件下，艰苦奋斗的传统决不丢，勤俭节约的要求永不忘，严格控制 “三公”经费，禁止新建楼堂馆所。加强公务员队伍建设，强化政治意识、责任意识和公仆意识,时刻保持蓬勃朝气、昂扬锐气、浩然正气，树立为民务实清廉的政府形象。

各位代表!百舸争流千帆竞发，奋勇当先时不我待。让我们在市委的坚强领导下，团结拼搏，开拓进取，务实重干，奋力前行，共同谱写漯河发展新篇章，为创造全市人民更加美好的幸福生活而努力奋斗!
